# Taxonomic and genomic attributes of oligotrophic soil bacteria

**DOI:** 10.1093/ismeco/ycae081

**Published:** 2024-06-12

**Authors:** Nicholas B Dragone, Michael Hoffert, Michael S Strickland, Noah Fierer

**Affiliations:** Cooperative Institute for Research in Environmental Sciences, University of Colorado Boulder, Boulder, CO 80309, United States; Cooperative Institute for Research in Environmental Sciences, University of Colorado Boulder, Boulder, CO 80309, United States; Department of Ecology and Evolutionary Biology, University of Colorado Boulder, Boulder, CO 80309, United States; Department of Soil and Water Systems, University of Idaho, Moscow, ID 83844, United States; Cooperative Institute for Research in Environmental Sciences, University of Colorado Boulder, Boulder, CO 80309, United States; Department of Ecology and Evolutionary Biology, University of Colorado Boulder, Boulder, CO 80309, United States

**Keywords:** bacterial oligotrophy, copiotrophy, carbon limitation, soil bacteria, rhizosphere, bacterial traits, bacterial life history strategies

## Abstract

Not all bacteria are fast growers. In soil as in other environments, bacteria exist along a continuum—from copiotrophs that can grow rapidly under resource-rich conditions to oligotrophs that are adapted to life in the “slow lane.” However, the field of microbiology is built almost exclusively on the study of copiotrophs due, in part, to the ease of studying them *in vitro*. To begin understanding the attributes of soil oligotrophs, we analyzed three independent datasets that represent contrasts in organic carbon availability. These datasets included 185 samples collected from soil profiles across the USA, 950 paired bulk soil and rhizosphere samples collected across Europe, and soils from a microcosm experiment where carbon availability was manipulated directly. Using a combination of marker gene sequencing and targeted genomic analyses, we identified specific oligotrophic taxa that were consistently more abundant in carbon-limited environments (subsurface, bulk, unamended soils) compared to the corresponding carbon-rich environment (surface, rhizosphere, glucose-amended soils), including members of the Dormibacterota and Chloroflexi phyla. In general, putative soil oligotrophs had smaller genomes, slower maximum potential growth rates, and were under-represented in culture collections. The genomes of oligotrophs were more likely to be enriched in pathways that allow oligotrophs to metabolize a range of energy sources and store carbon, while genes associated with energy-intensive functions like chemotaxis and motility were under-represented. However, few genomic attributes were shared, highlighting that oligotrophs likely use a range of different metabolic strategies and regulatory pathways to thrive in resource-limited soils.

## Introduction

In 1991, A.M. Semenov described oligotrophic microorganisms as those “that are evolutionarily adapted to exploit ecological niches characterized by low substrate concentrations and low energy flow” [1]. Compared with copiotrophs that can grow rapidly in carbon-rich environments, oligotrophs instead rely on efficient resource use to survive in environments where the substrates required to fuel growth and metabolism are in limited supply [2–5]. Dividing bacteria into categories based on general life history strategies is not easy, and oligotrophs are no exception. Rather than representing a discrete category, heterotrophic bacteria span a continuous gradient from more copiotrophic to more oligotrophic lifestyles [6–9]. Previous studies have mainly focused on aquatic systems, where Lauro *et al*. [10] have estimated that oligotrophs dominate. Studies have also been performed in soil systems to attempt to identify where specific bacterial taxa and/or lineages fall along the oligotrophic to copiotrophic spectrum [9]. However, the specific traits and genomic attributes that differentiate soil bacteria across this spectrum remain largely undetermined.

Oligotrophic bacteria should be more dominant in soil environments with lower concentrations of available organic C [9, 11]. We therefore expect that oligotrophs are more abundant in bulk than in rhizosphere soils [12], deeper than shallower soils [13], and surface soils in systems with low plant net primary productivity compared to systems with greater plant-derived organic C [14]. However, even soils with high concentrations of organic matter could favor oligotrophs if that organic matter is unavailable to fuel microbial metabolism, either due to chemical recalcitrance, physical protection, or other factors that make organic carbon resistant to microbial catabolism [15].

We expect that soil environments dominated by more oligotrophic bacteria are common. As one line of evidence, consider that 35% to 50% of the microbial biomass contained in soils is located in subsurface horizons that generally have lower levels of available organic carbon compared to surface soils [13, 16–20]. Likewise, even at the scale of individual bacterial cells, most of the available surface area in soil is not occupied [21]. Finally, consider that the generation times of soil bacteria are quite long, on the order of weeks [22], highlighting that conditions which we would expect to favor oligotrophic soil bacteria are likely the norm, not the exception.

We note that the amounts of available organic substrates are not the only factor limiting microbial growth in soil; there are abiotic stressors (e.g. low pH, moisture limitation, anaerobic conditions) and disturbances (e.g. predation, drying–rewetting and freezing–thawing events) that can also limit microbial growth, even in soils where substrate concentrations are high [23, 24]. Thus, soils that favor oligotrophic bacteria due to reduced substrate availability can also be environments that might favor bacteria tolerant of other abiotic or biotic stressors or disturbances. To give one example, hyper-arid desert soils in Antarctica and the Atacama Desert typically have low inputs of plant-derived organic C, but the microbes living in desert soils also have to tolerate conditions of low moisture, high ultraviolet exposure, and high soluble salt concentrations [25, 26]. Oligotrophic bacteria, by definition, must be able to tolerate environments where organic substrate availability is limited, but they may also have to tolerate other conditions that could simultaneously act to constrain growth in such environments.

There has been a rapid increase in the availability of genomic data from a broad diversity of bacteria, making genomic analyses an important strategy to infer the traits and attributes of bacteria, especially when coupled with cultivation-based assessments of phenotypes. However, neither genomic information nor well-characterized isolates are currently unavailable for many soil bacterial taxa, even abundant and ubiquitous taxa [24, 27]. We would expect oligotrophs to be particularly under-represented in pre-existing genome databases and culture collections given that they are likely difficult to cultivate using standard approaches which typically favor fast-growing taxa that can thrive on rich media [2, 5, 28]. While some oligotrophs can be cultivated, most notably demonstrated through the cultivation of SAR11 from marine waters using extremely dilute media and long incubation periods [29, 30], doing so is neither easy nor quick. The under-representation of oligotrophic bacteria in pre-existing culture collections has two important ramifications. First, it means that the physiological attributes of oligotrophs have not been as well characterized as those of more copiotrophic taxa which are more amenable to *in vitro* study [11]. Second, it means that pre-existing genomic databases will be biased against soil oligotrophs as most high-quality bacterial genomes are obtained from the sequencing of cultured isolates. For example, 70% of the bacterial genomes in one of the largest curated genome databases, Genome Taxonomy Database (GTDB), are currently from isolates [31]. For these reasons, it has remained difficult to identify the genes, or gene categories, that may be characteristic of soil oligotrophic bacteria and what those genomic attributes could tell us about the physiological adaptations of oligotrophic bacteria.

What are the expected traits of soil bacterial oligotrophs? Oligotrophic bacteria that are heterotrophs should be able to survive and grow under conditions where metabolizable organic substrates are infrequently supplied and/or supplied at consistently low concentrations [1, 2, 4, 5]. We would expect that bacteria able to thrive under such soil conditions might share similar ecological attributes. Previous research from marine systems have identified certain phenotypic traits that have long been thought to be characteristic of oligotrophic soil bacteria including (but are not limited to) long generation times, low maximal specific growth rates (*μ*_max_), low maintenance energy requirements, high substrate uptake affinities, ability to accumulate intracellular storage polymers, smaller cell sizes (high surface area/volume ratios), and higher density of transport sites per unit cell surface area (and/or low specificity transporters) [1–4, 10, 32–35]. Most of these hypotheses regarding oligotrophy-associated traits are supported by limited evidence and there is considerable uncertainty regarding the validity of these hypotheses. For example, Noell and Giovannoni [33] proposed that small genome size is associated with oligotrophs, while Vieira-Silva and Rocha [32] have argued otherwise. A more detailed list of 18 hypothesized genes, gene categories, or other genomic features that might be associated with more oligotrophic soil bacteria, based on pre-existing work focused on soil bacteria and on heterotrophic bacteria found in other environments (including marine environments), can be found in Table 1.

**Table 1:** Genomic characteristics (A), functional gene categories (B), and individual genes (C) that have been hypothesized in previous studies as being indicative of oligotrophic bacteria.

**A. Genomic characteristic**	**Hypothesis**	**Reference**
Delta ENC	*Codon usage bias in highly expressed genes (a proxy for maximum potential growth rate) should be lower for oligotrophs*	[32]
Estimated rRNA operon copy #	*Oligotrophs*, *with lower maximum potential growth rates*, *should have fewer rRNA operons*	[3, 32]
Genome size	*Oligotrophs have smaller genomes*	[10]
**B. Functional categories**		
Amino acid transport and metabolism	*Oligotrophs should have more genes associated with amino acid transport and metabolism to facilitate the enhanced utilization of proteinaceous substrates*	[36]
Chemotaxis and motility	*Sensing and moving is an energetically expensive foraging strategy and should be less common in oligotrophs*	[3, 10]
Lipid transport and metabolism	*Oligotrophs are expected to be enriched in lipid transport and metabolism genes for C storage*	[10]
Secondary metabolite biosynthesis, transport, metabolism	*Oligotrophs may have more genes associated with secondary metabolite metabolism*	[10]
Defense mechanisms	*Oligotrophs should have fewer genes allocating energy to defense*	[37]
Transcription	*Oligotrophs should have fewer genes allocated to transcription*	[37]
Signal transduction	*Oligotrophs should have fewer genes allocated to signal transduction*	[37]
Cellular replication, recombination, repair	*Oligotrophs should have fewer genes and allocate less energy to cellular replication*, *recombination*, *and repair*	[4]
**C. Specific genes**		
Glycine betaine ABC transporter (ProX)	*Glycine betaine ABC transporters are more abundant in oligotrophs*	[33]
RNA polymerase, extracytoplasmic E (rpoE)	*Transcription factor involved in environmental stress responses should be more common in oligotrophs*	[10]
Trehalose synthase and transporter	*Universal stress molecule and osmolyte that stabilizes proteins* are *expected to be more common in oligotrophs*	[34]
Form 1 CO dehydrogenases (coxL)	*Consumption of CO*, *even at low concentrations, is beneficial for oligotrophs and genes associated with this metabolic pathway will be more common*	[38]
[NiFe] hydrogenases	*Genes involved in H_2_ metabolism*, *which can serve as an energy source in challenging environments*, *should be more common in oligotrophs*	[39, 40]
		
Thiamine biosynthesis	*Genes related to thiamine biosynthesis should be less common in oligotrophs*, *who gather thiamine from exogenic sources*	[3]
Poly-B-hydroxybutyrate, polyhydroxyalkanoate	*Oligotrophs should have more genes associated with poly-B-hydroxybutyrate and polyhydroxyalkanoate synthesis to cope with periods of starvation*	[2]

Here, we analyzed three independent datasets to test hypotheses about oligotrophic versus copiotrophic soil bacteria. These include a “soil profile” dataset of samples collected from 20 soil profiles representing distinct soil and ecosystem types across the USA [13] and a “rhizosphere” dataset of paired bulk soil and rhizosphere samples collected from a range of plant species and locations across Europe [41]. For the soil profiles, we used depth as a proxy for carbon availability because most fresh C inputs are derived from plant litter and root exudates with soil microbial biomass, SOC, and respiration rates declining sharply with depth [42–46]. We also expected the rhizosphere samples to have more available carbon than the corresponding bulk soils due to root exudates and rhizodeposition, as has been shown previously [12, 47–49]. Thus, we presume that these field datasets capture site-specific contrasts in organic C availability (surface vs. subsurface soils, and rhizosphere vs. bulk soils); however, we recognize that organic C availability is not the only factor that can vary with depth or with proximity to plant roots. Thus, we also included a third dataset where soil organic C availability was experimentally manipulated in the absence of other potential confounding factors, amending soil microcosms with glucose over a 4-month period [50]. Using DNA sequence information from these three datasets, we identified the bacteria that consistently have higher relative abundances in soils with more available C (surface, rhizosphere, glucose-amended soils) versus soils that are likely more C limited (subsurface, bulk, unamended soils). Specifically, we aimed to identify putatively oligotrophic bacterial taxa and to test the hypotheses outlined in Table 1 regarding the genomic attributes previously hypothesized to be associated with oligotrophic bacterial heterotrophs.

## Materials and methods

### Sample collection and data acquisition

Details regarding the soil sampling process and characterization of samples included in the “soil profile” dataset are provided in Brewer *et al*. [13]. In brief, they collected 185 soil profile samples from 10 different Critical Zone Observatories across the USA. Two soil profiles representative of distinct soil types found at each Critical Zone Observatory site were sampled in 10-cm increments to 100 cm in depth or to refusal. They then conducted marker gene sequencing using the 515f/806r primer pair to allow for sequencing of the V4–V5 region of the 16S rRNA gene. We downloaded their raw 16S rRNA gene sequencing data from Figshare at https://doi.org/10.6084/m9.figshare.4702711.

Details regarding the sampling process and characterization of the soils included in the “rhizosphere” dataset can be found in Ramirez *et al*. [41]. To summarize, they collected paired rhizosphere samples and bulk soil samples from under and around range-expanding plants across six countries in Europe. Their 950 samples were also sequenced using the primer pair 515f/806r. We accessed the raw 16S rRNA gene sequences from the European Nucleotide Archive under accession number PRJEB25694 for bulk soils and PRJEB25692 for rhizosphere soils.

Details of the experimental design used for the “microcosm” samples are described in Lucas *et al*. [50]. To summarize, they created microcosms (50 g of dry weight soil) from sub-samples of a single homogenized surface soil (1–10 cm depth) collected from a mixed deciduous forest in Virginia, USA. Weekly additions of glucose (260 μg C g dry wt soil^−1^ day^−1^) were added to four of the microcosms over a 117-day period, with five of the microcosms receiving only an equivalent amount of water (no glucose) over the same 117-day period [50]. DNA extraction and amplicon sequencing methods for these samples are described in Ramoneda *et al*. [51]. Raw 16S rRNA sequence from these nine “microcosm” samples were downloaded from the European Nucleotide Archive (accession number PRJNA1071192).

### Taxonomic analysis via amplicon sequencing

We processed the 16S rRNA gene sequences from the 185 “soil profile” samples, the 950 “rhizosphere” samples, and the 9 “microcosm” samples using the DADA2 pipeline v.1.26 [52]. All three datasets were processed independently. For each dataset, sequences were quality filtered and clustered into amplicon sequence variants (ASVs, 100% sequence similarity), with taxonomy determined using a naïve Bayesian classifier method [53] trained against the SILVA reference database v.138 [54, 55]. A minimum bootstrapping threshold required to return a taxonomic classification of 50% similarity was used for analysis. More details of the specific parameters used can be found at https://github.com/fiererlab/dada2_fiererlab. Raw ASV tables for each of the three datasets can be found in Dataset S1.

For the soil profile dataset, we removed samples that did not have >10 000 reads (7 samples) which left us with 178 samples in total, 139 subsurface (>20 cm depth) and 39 surface soils (0–20 cm depth). ASVs associated with chloroplast, mitochondria, and eukaryotes (785 ASVs total) as well as those unassigned to the phylum level (613 ASVs) were removed. As we were most interested in the more abundant and ubiquitous taxa, ASVs with <50 reads across all 178 samples were removed (21 570 ASVs) and ASVs that were found in <5 profiles were also removed (6012 ASVs). A total of 12 075 ASVs remained for downstream analyses.

For the bulk soil and rhizosphere dataset, we only included the 929 samples that had >1000 reads for downstream analyses (443 bulk soil and 486 rhizosphere samples). ASVs associated with chloroplasts, mitochondria, and eukaryotes (2856 ASVs total) as well as those unassigned to the phylum level (780 ASVs) were then removed. As with the soil profile dataset, ASVs with <50 reads across all 929 samples were removed (27 837 ASVs) and ASVs that were found in <5 samples were also removed (6533 ASVs), leaving a total of 9638 ASVs for downstream analysis.

For the nine samples from the “microcosm” experiment, ASVs associated with chloroplasts, mitochondria, eukaryotes (213 ASVs total) as well as those unassigned to the phylum level (26 ASVs) were removed. ASVs with <50 reads across the nine samples were removed (1288 ASVs) as were ASVs that were found in fewer than three samples (162 ASVs). This left a total of 726 ASVs for downstream analysis.

To determine which bacterial taxa are more likely to be found in the subsurface soils (*n* = 39) versus the surface soils (*n* = 139), in the bulk soils (*n* = 486) versus the rhizosphere soils (*n* = 443), and in the unamended soils (*n* = 5) versus the glucose-amended soils (*n* = 4), we used Mann–Whitney nonparametric tests corrected for multiple comparisons with Bonferroni tests to compare the relative abundance of each ASV within each individual dataset. The relative abundance of each ASV in each sample was calculated by dividing the number of reads assigned to that ASV by the total number of reads for a sample remaining after the filtering described above. Bacterial ASVs that were significantly more abundant in the subsurface soil samples (178 ASVs) were classified as “subsurface soil-associated” and considered more likely to be oligotrophic, while those significantly more abundant in surface samples (1271 ASVs) were considered to be more copiotrophic. Similarly, bacterial ASVs that were significantly more abundant in the bulk soils (2779 ASVs) were considered more oligotrophic while those that were significantly more abundant in rhizosphere samples (1366 ASVs) were considered more copiotrophic. Finally, bacterial ASVs that were significantly more abundant in the unamended (no glucose) microcosms (169 ASVs) were considered more oligotrophic while those that were significantly more abundant in the glucose-amended microcosms (239 ASVs) were considered more oligotrophic. In total, 8658 ASVs showed no significant difference in abundance between the subsurface and surface soils, 5434 ASVs showed no significant difference in abundance between the bulk and rhizosphere soils, and 314 ASVs showed no significant difference in abundance between the no glucose and glucose soils. A summary of all bacterial ASVs associated with each category per dataset can be found in Dataset S2. While 0.20%–3.40% of the ASVs detected across the three datasets were identified as archaeal, we focus just on bacteria for this study.

The sequences of the 1449 ASVs that we identified as either being subsurface- or surface soil-associated (178 ASVs and 1271 ASVs respectively) and the 4145 ASVs we identified as either bulk soil- or rhizosphere-associated (2779 ASVs and 1366 ASVs respectively) and the 408 ASVs identified as either associated with the “no glucose” microcosm soils or the glucose-amended soils (169 ASVs and 239 ASVs respectively) were matched against the GTDB release 207 [31, 56, 57] using VSEARCH v2.22.1 (--strand both --notrunclabels --iddef 0 --id 0.97 --maxrejects 100 --maxaccepts 100) [58]. If a single ASV matched to multiple GTDB genomes equally, the most complete genome with the lowest contamination was chosen as the reference. A total of 453 surface-associated ASVs and 66 subsurface soil-associated ASVs matched to reference genomes in GTDB (Fig. S1). Before any additional analyses were performed, we removed reference genomes that matched to both categories (surface and subsurface soils) which yielded a total of 40 unique subsurface soil genomes and 303 unique surface soil genomes (Fig. S2, Dataset S3). For the “rhizosphere” dataset, a total of 825 bulk soil-associated ASVs and 782 rhizosphere-associated ASVs matched to reference genomes in GTDB (Fig. S1), giving us a total of 336 unique bulk soil genomes and 592 unique rhizosphere genomes (Fig. S2, Dataset S3). Finally, for the “microcosm” dataset, a total of 80 ASVs from the unamended samples and 97 ASVs from the glucose-amended samples matched to reference genomes in GTDB (Fig. S1), giving us a total of 66 and 71 unique genomes for the unamended and glucose-amended treatments, respectively (Fig. S2, Dataset S3).

### Genomic analyses of representative genomes

General characteristics of the 1408 genomes representative of the inferred copiotrophs and oligotrophs from the three independent datasets were compiled from the metadata associated with the GTDB reference database release 207 [31, 57]. More specifically, we used information about the genome category (metagenome assembled genome (MAG) vs. isolate), predicted genome size, GC percentage, predicted small subunit rRNA gene count, and taxonomy (based on SILVA reference database v.138 [54, 55]) (Dataset S3). For more information about how the metadata were generated by GTDB, see details in Parks *et al*. [31, 56] and https://gtdb.ecogenomic.org/methods.

To estimate the predicted maximum potential growth rate for each of the 1408 reference genomes, we used the tool gRodon2 on the genome scaffolds downloaded from GTDB following the authors’ recommendations for MAGs and genomes as detailed in Weissman *et al*. [59] and https://github.com/jlw-ecoevo/gRodon. The gRodon2 tool estimates maximal growth rates from codon usage biases in highly expressed genes, an indicator of selection for rapid growth [32, 59]. We note that gRodon2 only provides a prediction of maximum potential growth rates, not actual growth rates, and the calculated values are simply estimates useful for inferring broad patterns in maximum potential growth rates across genomes [59].

To determine the functional gene abundances in each reference genome, we used the blastp function of DIAMOND v.2.0.15 (-k 1 -e 10-10 --query-cover 90) [60] to annotate the 1408 genomes against the database of Clusters of Orthologous Genes (COGs) ontology v.2020 [61, 62]. For calculating the abundances of the COGs, COG categories, and COG groups listed in Table 1, we normalized the reads assigned to each of the 4877 individual COGs by the estimated genome size for each reference genome and gene abundances are presented as reads per million base pairs. The abundances of COGs associated with different hypotheses were determined by summing the normalized gene abundances of each COG or COG category associated with the specific hypothesis being tested (Table 1, Table S1). For the 25 COG categories, we also followed a method used by Weissman *et al*. [59]. Briefly, we calculated the proportion of COGs associated with each category by dividing the number of genes assigned to that category by the total number of COGs identified. If a COG was found to be assigned to multiple categories, it was counted in each. The abundance of each COG in each sample can be found in Dataset S4.

To determine differences in the genomic characteristics, predicted maximum growth rates, abundance of COGs, COG categories, and specific functional genes, we used Mann–Whitney nonparametric tests corrected for multiple comparisons with Bonferroni tests to compare the presence and abundance of these features between subsurface soil-associated genomes (40 genomes) and surface soil-associated genomes (303 genomes), between the bulk soil genomes (336 genomes) and the rhizosphere genomes (596 genomes), and between the genomes from the unamended microcosms (66 genomes) and the glucose-amended microcosms (71 genomes). Functional genes and gene categories that were significantly more abundant in subsurface, bulk, and unamended soils were considered to be oligotroph-associated traits while those significantly more abundant in surface soil, rhizosphere soil, and glucose soil genomes were considered to be copiotroph-associated traits.

### Plotting and additional analysis in R

Supporting analyses were performed in R v.4.2.2 [63]. Statistical tests were performed using the base R functions “wilcox.test” and the packages “rstatix” (https://github.com/kassambara/rstatix). Plotting was performed using the R packages “ggplot2” and “cowplot.” ASV table filtering, stacked bar plots of relative abundance, and other ASV-based analyses were performed using the R package “mctoolsr” (https://github.com/leffj/mctoolsr/).

## Results and discussion

### Evidence of gradients in organic C availability

We used three independent datasets that we expect to each represent categorical differences in organic C availability and thus differences in the predominance of oligotrophic bacteria. For the “soil profile” dataset (185 soils from 20 profiles across the USA; see Brewer *et al*. [13]), we first divided the samples into those coming from subsurface horizons (20 to 100 cm in depth) and surface horizons (top 20 cm). As expected, total organic C concentrations were significantly higher in the surface soils than in the subsurface soils (Fig. S3), with surface soils having, on average, 3.6 times higher organic C concentrations. While we did not quantify the fraction of the organic C available to microbes, we would expect that the bioavailability of organic C also decreases with depth [20, 44, 64].

We recognize that there are other abiotic factors that also vary with soil depth, including, but not limited to, the availability of other nutrients (including N, P), moisture, and temperature. However, analyses of these same soils [13, 65] have shown that other soil variables (including pH) exhibit minimal consistent changes with soil depth. Notably, Dove *et al*. [65] also found that both extracellular enzyme activity and microbial biomass concentrations decrease with depth across these samples, further evidence that soil profiles represent a gradient in organic C availability, with deeper soils being more organic carbon limited and more likely to harbor oligotrophic bacteria as compared to surface soils.

For the “rhizosphere” dataset, we had 950 paired samples of bulk and rhizosphere soils collected from a wide range of plant species and locations across Europe; see Ramirez *et al*. [41] for details. Data on organic C concentrations were not available for these samples, but we expect more organic carbon to be available to microbial communities in the rhizosphere. Plant-derived inputs of organic carbon into rhizosphere soils has been well described [47, 66] and root-associated C fluxes have been shown to be a major contributor to soil C pools [67] with rhizosphere carbon inputs representing 30%–40% of total carbon inputs to soil, despite the rhizosphere being <1% of the world’s total soil volume [48]. We also know that the input of C from root exudates can substantially increase microbial biomass and activity in the rhizosphere compared to bulk soils [68, 69]. We recognize that the specific amounts and quality of organic carbon inputs to the rhizosphere will depend on plant species, plant age, soil texture, and other factors [70], but previous studies have consistently found evidence that supports our designation of bulk soils as being more C limited than rhizosphere soils (see refs [12, 47–49] for examples). However, we also recognize that there are likely other biotic and abiotic factors, in addition to soil C availability, that can differ between bulk and rhizosphere soils.

Given that organic C availability is not the only factor that differs between surface and subsurface soils, or between bulk and rhizosphere soils, we also included a third dataset that represents a direct experimental manipulation of available C in laboratory microcosms, with other factors held constant. For this “microcosm” study, four replicate microcosms containing a single soil type were incubated for 4 months during which they received weekly additions of glucose, with five microcosms containing the same soil and incubated under identical conditions, but without any glucose added. By comparing changes in microbial communities across the three independent datasets, we can identify taxa, and the traits of those taxa, that consistently differ between soils that we would expect to favor more oligotrophic soil bacteria over more copiotrophic soil bacteria.

### Taxa consistently associated with soil carbon availability

Bacteria span a continuous gradient from more copiotroph to more oligotrophic lifestyles [6, 7], and our data support our initial hypothesis that oligotrophy is challenging to predict from taxonomy alone. Our results are in line with previous work [9] highlighting that many taxonomic groups, especially at broader levels, can include both more-oligotrophic and more-copiotrophic members.

**Figure 1:**
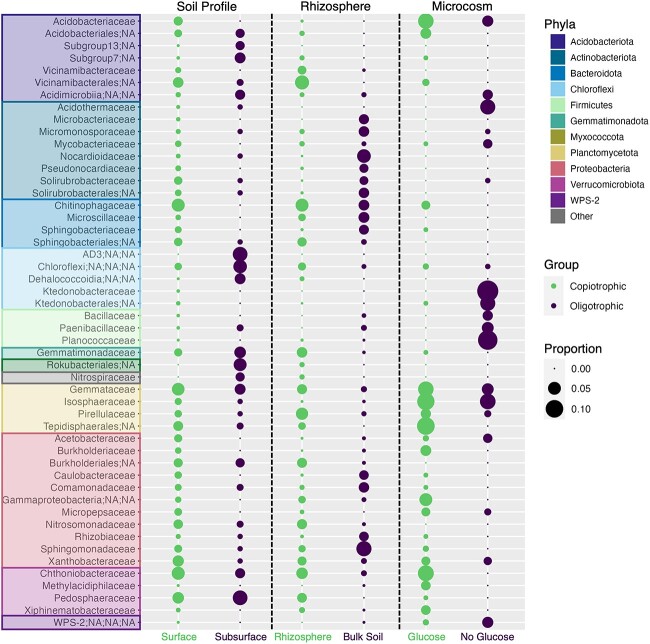
Proportion of ASVs inferred to be either copiotrophic or oligotrophic and their taxonomic affiliation at the family level. Results are presented for each of the three datasets that were analyzed separately (with results from each dataset separated with the dashed vertical lines). For the “soil profile” dataset (left column), we identified 1271 ASVs enriched in the surface soils and 178 ASVs enriched in the subsurface soils. For the “rhizosphere” dataset (center column), we identified 2838 ASVs enriched in bulk soils and 1366 ASVs in the associated rhizosphere soils. For the “microcosm” dataset (right column), we identified 239 ASVs enriched in the glucose-amended microcosms and 169 ASVs enriched in the unamended (no glucose) microcosms. See Dataset S2 for specific details on the ASVs associated with each group. Families were only included if they made up at least 2% of the relative abundance of at least one sample category. The size of the bubbles indicates the proportion of the total number of ASVs assigned to that taxonomic group. Bubbles are colored based on whether they were identified as being associated with more carbon-rich (rhizosphere, surface soils, glucose-amended microcosms) or more carbon-limited environments (bulk soil, subsurface soils, unamended microcosms).

For the “soil profile” dataset, we compared the abundances of the 12 075 ASVs (100% sequence identity) recovered from the 16S rRNA gene sequencing effort conducted across all 20 soil profiles, comparing surface versus subsurface soils. We identified 178 bacterial ASVs that were consistently more abundant in subsurface soils and 1271 ASVs that were consistently more abundant in surface soils (Fig. 1, Dataset S2). There were more distinct families associated with the surface than the subsurface (159 and 82 families, respectively), a result that is to be expected given that we found nearly seven times more surface-associated taxa than subsurface-associated taxa. While the bacterial families that were most abundant in the subsurface soils (Pedosphaeraceae, unidentified Chloroflexi, unidentified Rokubacteriales, Gemmatimonadaceae) were different from those that were more abundant at the surface (Chthoniobacteraceae, Chitinophagaceae, Gemmataceae, Pedosphaeraceae, Xanthobacteraceae; see Fig. 1 for more details), 31% of the families identified by this analysis (62 of the 202 total) included ASVs assigned to both the subsurface- and surface-associated groups, highlighting that presumably copiotrophic and oligotrophic bacterial taxa can be found within related groups, a result in line with other studies [9].

For the “rhizosphere” dataset, we were able to identify 2779 bacterial ASVs that were consistently more abundant in bulk soils and 1366 ASVs consistently more abundant in the rhizosphere soils using the same methods described for the soil profiles (Fig. 1, Dataset S2). Similar to the “soil profile” dataset, we found that 35% of the families identified in our analysis (108 of the 310 total families) included ASVs assigned to both the bulk soil- and rhizosphere-associated groups, though there were differences in the most abundant families between the two sample categories: Sphingomonadaceae, Nocardioidaceae, Microscillaceae, Chitinophagaceae, and Micromonosporaceae being consistently more abundant in bulk soils, and unidentified Vicinamibacterales, Chitinophagaceae, Pirellulaceae, Chthoniobacteraceae, and Gemmatimonadaceae being consistently more abundant in the rhizosphere soils (Fig. 1).

For the “microcosm” dataset, we identified 169 ASVs that were consistently more abundant in the unamended (“no glucose”) microcosms and 239 ASVs that were consistently more abundant in the microcosms amended with glucose (Fig. 1, Dataset S2). Phyla consistently more abundant in the “no glucose” microcosms included Firmicutes (26.2% of ASVs), Chloroflexi (24.2%), Actinobacteriota (17.2%), Planctomycetota (14.2%), and Proteobacteria (10.1%) (Fig. S4). The taxa identified as being consistently over-represented in the glucose-amended microcosms were Planctomycetota (31.4% of glucose ASVs), Proteobacteria (22.2%), Acidobacteriota (19.7%), and Verrucomicrobiota (13.4%) (Fig. S4). Only 22% of the families identified in our analysis (19 of the 84 total families) included ASVs assigned to both microcosm treatments. The families that were more abundant in the “no glucose” microcosms were Ktedonobacteraceae, Planococcaceae, Isosphaeraceae, Acidothermaceae, and unidentified Ktedonobacterales, while unidentified Tepidisphaerales, Isosphaeraceae, Chthoniobacteraceae Acidobacteriaceae, and Gemmataceae were more abundant in the glucose-amended microcosms.

**Figure 2:**
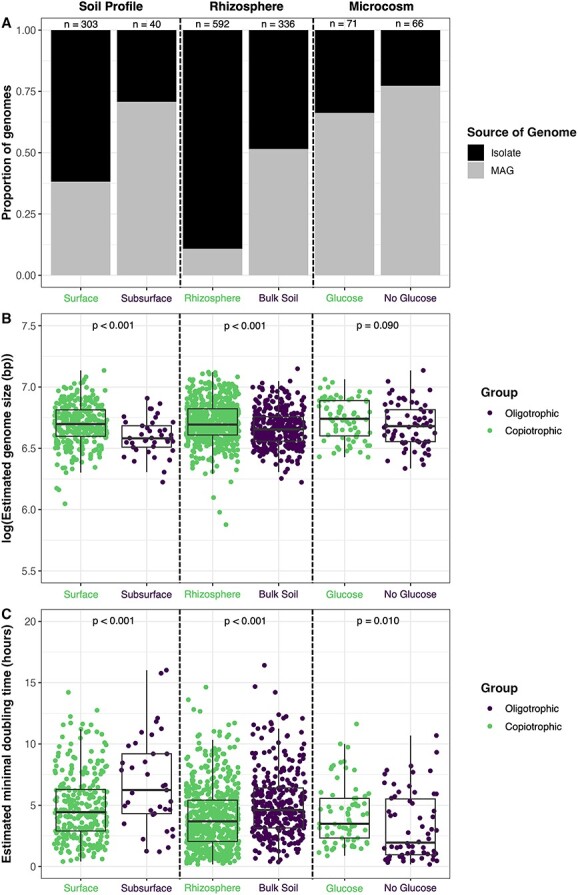
Characteristics of the genomes representative of bacterial taxa found to be indicative of soils with lower versus higher carbon availability for each of the three datasets (in columns, “soil profile” dataset on the left, “rhizosphere” dataset in the center, and the “microcosm” dataset on the right—see methods for details). The number of genomes per sample category are indicated at the top of the figure. (**A**) The origin of the representative genomes for each sample category, whether they were derived from metagenome-assembled genomes (MAGs) or from isolates. For each of the three datasets, the proportion of genomes derived from MAGs was higher than those derived from isolates in the lower carbon soil environment (oligotrophic) than in the corresponding higher carbon soil environment (copiotrophic). (**B**) Differences in genome sizes across the sample categories included in each dataset. Genome sizes were consistently smaller for representative taxa indicative of the lower C soil environments. (**C**) Estimated minimum doubling times of the representative genomes, as inferred using gRodon2 [59]. Minimum doubling times were significantly longer (lower maximum potential growth rates) in the subsurface soil genomes compared to surface soil genomes and in the bulk soil genomes compared to the rhizosphere genomes.

Together, our analyses suggest that, within a dataset, we can identify representative taxa that are more likely to be oligotrophic (associated with low-carbon soils) or copiotrophic (associated with high carbon soils). For example, in the “soil depth” dataset, we found that the putatively oligotrophic organisms associated with the subsurface, like Dormibacterota, have been described in other studies as having functional adaptations to survive in challenging, low-carbon environments [13, 71]. However, if we compare the putative oligotrophs (associated with subsurface, bulk, and “no glucose” soils) and copiotrophs (associated with rhizosphere, surface, and glucose-amended soils) across datasets, the patterns are more muddled. We did identify some families across the two datasets that exhibit consistent patterns; for example, Pedospheraceae, Ktedonobacteraceae, and unclassified Chloroflexi are more often associated with more carbon-limited soils, while Sphingomonadaceae, Chthoniobacteriaceae, and Solilrubrobacteraceae are more often associated with the more carbon-rich soils. However, many families show contrasting patterns across the three datasets. Members of the Chitinophagaceae were frequently identified as being more abundant in surface soils and glucose-amended soils, but members of this group did not exhibit differential abundances between the bulk and rhizosphere soils (Fig. 2). Similarly, members of the Gemmataceae, Sphingobacteriales, and Xanthobacteriaceae families were identified as putative oligotrophs and putative copiotrophs depending on the specific dataset in question (Fig. 2).

### Genomic features of oligotrophic soil bacteria

Given that taxonomic affiliation is not particularly useful for differentiating oligotrophic from copiotrophic taxa, we next sought to determine if there are genomic attributes that better differentiate these groups. By matching the sequences of the ASVs inferred to be more oligotrophic or more copiotrophic from each of the three datasets (see above and Fig. 1) against the GTDB [31, 56, 57], we compiled a dataset of 1408 representative genomes to test hypotheses about the genomic characteristics of oligotrophic soil organisms (Fig. S1, Dataset S3). For all comparisons described below, the representative genomes from the taxa inferred to be oligotrophic or copiotrophic were compared within each dataset (“soil profile,” “rhizosphere,” and “microcosm”) and not across datasets. For details on the numbers of genomes assigned to each category per dataset, see Fig. S1 and Dataset S3.

We found that more of the representative genomes from taxa inferred to be oligotrophic from the “soil profile” and “rhizosphere” datasets were MAGs (70% for subsurface, 51% for bulk soil) while more of the copiotrophic genomes were derived from cultivated isolates (62% for surface soils, 90% for rhizosphere soils) (Fig. 2A). This same pattern held for the “microcosm” dataset, where MAGs constituted a greater percentage (77.3%) of the putatively oligotrophic genomes identified as being more abundant in the “no glucose” microcosms as compared to the representative genomes from taxa enriched in the glucose-amended microcosms (66.2%) (Fig. 2A). The genomes of bacteria inferred to be oligotrophs were more often generated through cultivation-independent methods than the genomes of copiotrophs, a pattern expected given the well-recognized challenges of cultivating and isolating oligotrophic taxa [29, 30, 72], further highlighting that cultivation-based methods are biased toward more copiotrophic taxa [4].

We found no significant difference in GC content between the representative copiotrophic and oligotrophic genomes from the “soil profile” dataset (Mann–Whitney *U*, *P* = .18), but we did find GC content to be significantly higher in copiotrophic genomes from the “rhizosphere” dataset (Mann–Whitney *U*, *P* < .001) and “microcosm” dataset (Mann–Whitney *U*, *P* = .01) (Fig. S2). Estimated genome sizes were significantly smaller for oligotrophic taxa in the “soil profile” and “rhizosphere” datasets with a similar pattern observed for the “microcosm” dataset (though this pattern was not significant, *P* = .09, Fig. 2B). A “streamlined” genome, or reduced genome size with fewer genes, has been presumed to be associated with oligotrophic bacteria reflecting reduced metabolic costs [28, 73, 74]. As genome reduction has also been linked to symbiotic microbial taxa [75], pathogens [76], and other ecological strategies besides oligotrophy, we are hesitant to conclude that a smaller genome size is a robust indicator of bacteria with a more oligotrophic life history strategy. Likewise, we found that estimated minimal doubling times (as inferred from analyses of codon usage bias; see ref [59]) were significantly longer for genomes of oligotrophic taxa than for genomes of copiotrophic taxa in two of the three datasets (Fig. 2C), supporting our hypothesis (Table 1).

### Functional attributes of oligotrophic soil bacteria

**Figure 3:**
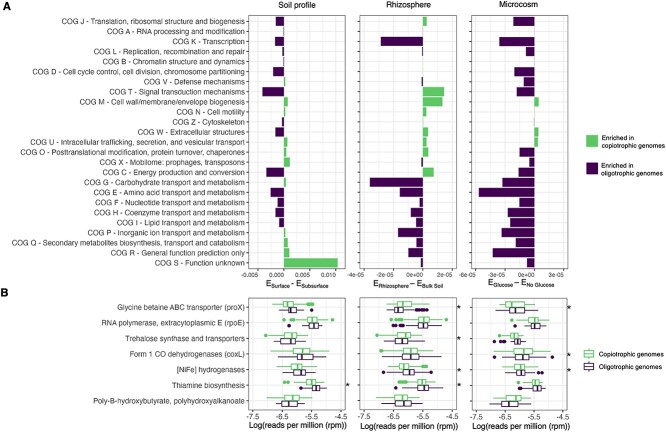
Comparisons of the abundances of 33 gene (COG) categories between the genomes representative of taxa indicative of the subsurface (*n* = 40) and surface soils (*n* = 303), between the bulk soils (*n* = 336) and rhizosphere soils (*n* = 592), and between the unamended (no glucose, *n* = 66) and glucose-amended (*n* = 71) microcosms. (**A**) Differences in the average proportion of genes in the more copiotrophic group (*E*_copiotrophic_) and the average proportion of genes in the more oligotrophic group (*E*_oligotrophic_) for each dataset pair. Negative values indicate gene categories enriched in oligotrophic genomes while positive values indicate gene categories enriched in copiotrophic genomes are colored based on which group has a higher proportion of those genes (i.e. whether the gene category was enriched in oligotrophic genomes or copiotrophic genomes). (**B**) Abundances of genes associated with the hypotheses outlined in Table 1C. Significant differences in gene abundances between the sample categories within a given dataset are starred (Mann–Whitney *U*, *P* < .05). Information on individual COGs can be found in Dataset S4.

We next used the “soil profile,” “rhizosphere,” and “microcosm” datasets to test 15 specific hypotheses compiled from the literature regarding the functional attributes of oligotrophs (see Table 1B,C). We compared the abundances of genes associated with 25 COG (clusters of orthologous genes) functional categories between the representative genomes from the inferred copiotrophic and oligotrophic taxa identified from each dataset. We found that the categories that were enriched in genomes from more oligotrophic taxa supported several of the hypotheses outlined in Table 1 (Fig. 3). For example, the representative genomes from taxa inferred to be more oligotrophic had more genes associated with amino acid transport and metabolism (COG E) across all three datasets. These genes facilitate the enhanced metabolism of proteinaceous substrates which is thought to be particularly beneficial in resource-limited environments [36]. We also found that oligotrophic genomes had a wider range of metabolic pathways related to lipid transport and metabolism, functions that have been linked to bacterial C storage (Fig. 3). In contrast, copiotrophic genomes were enriched in genes associated with energetically costly activities, including chemotaxis and motility, intracellular trafficking, secretion, and vesicular transport, and cell wall biogenesis (Fig. 3) [3, 10, 37]. However, not all the results from our comparative genomic analyses supported the hypotheses outlined in Table 1. From previous work, we would expect that oligotrophs have fewer genes associated with transcription [37], but we found that genes associated with transcription were more abundant in the representative genomes of oligotrophic taxa (Fig. 3A). Notably, we also found many gene categories that exhibited inconsistent patterns across the three datasets. For example, we found that genes associated with secondary metabolite biosynthesis, transport, and metabolism (COG Q) were enriched in copiotrophs from the surface soils, in contrast to expectations [10], but these same genes were enriched in the representative oligotrophic genomes from the other two datasets (Fig. 3A). We observed the same inconsistent patterns across the three datasets for several other COG categories (including P, R, S, and X) (Fig. 3A).

When we examined more specific genes and functions that have previously been hypothesized to be associated with oligotrophs (Table 1C), we found just one hypothesis supported by consistent patterns observed across all three datasets. [NiFe] hydrogenase genes, which are involved in trace gas metabolism of H_2_ [38, 39, 77, 78], were more abundant in genomes from oligotrophs across all three datasets. The ability to oxidize H_2_ has previously been shown to be widespread across soil bacteria [40] and particularly important for bacterial survival and growth in resource-limited soil environments, including hyper-arid desert soils [26] and very young soils found on a newly formed volcanic island [79], results that are consistent with our findings. Genes for the metabolism of other trace gases do not necessarily show the same pattern. For example, Form 1 CO dehydrogenases, which facilitate the metabolism of CO, were, on average, more abundant in copiotrophs (Fig. 3).

To summarize, we tested specific hypotheses (Table 1) by comparing gene abundances between taxa inferred to be copiotrophic from those inferred to be oligotrophic. Although we did find some support for specific hypotheses given that the expected patterns were consistent across all three datasets, as noted above, the differences in gene abundances between inferred copiotrophs and oligotrophs were often negligible or inconsistent across datasets (Fig. 3). Together, these results highlight that observations from a single dataset may not apply more generally. Likewise, some of the hypotheses regarding the genomic features that distinguish copiotrophic from oligotrophic taxa (hypotheses often derived from the study of a relatively narrow range of bacterial diversity, Table 1) may not be robust when considering the broad diversity of bacteria found in soil.

### Hypotheses about oligotrophic bacteria

To further identify other genomic attributes that may be associated with oligotrophic bacteria, we next performed an untargeted search for any individual COGs that were consistently more abundant in the putatively oligotrophic bacterial genomes across all three datasets. We found 103 COGs that were significantly more abundant in the oligotrophic genomes and 14 COGs that were significantly more abundant in the copiotrophic genomes (Dataset S5). The other remaining 4760 COGs were either not significantly different between the groups or exhibited differing patterns across the datasets.

We found that over 50% of the individual COGs identified as more abundant in the genomes of oligotrophic taxa were assigned to six functional categories: translation, ribosomal structure, and biogenesis (J); signal transduction mechanisms (T); energy production and conversion (C); post-translational modification, protein turnover, and chaperones (O); amino acid transport and metabolism (E); and COGs with general function prediction only (R). In contrast, the only COG categories that had more than one COG identified as being more abundant in the genomes of copiotrophic taxa were COG category L (replication, recombination, repair, 2 COGs) and COG category S (function unknown, 6 COGs) (Dataset S5). While these patterns further reveal the propensity for oligotrophs to have a wide diversity of genes associated with energy acquisition, as described in [10], it is noteworthy that we observed oligotrophs to have more genes associated with translation and post-translational processes. Recent work in marine environments has shown that marine oligotrophs in resource-limited environments compensate for a lack of transcriptional regulation genes with a greater number of genes associated with post-translational mechanisms that modify proteins and result in changes in enzyme function [8, 80, 81]. More specifically, we see that many of the COGs that are more abundant in oligotrophic taxa are assigned to genes associated with tRNA modifications (e.g. COG0336: trmD, COG0820: rlmN, COG0343: tgt, see Dataset S5). Post-transcriptional tRNA modification has been found to be a moderator of cellular stress responses in prokaryotes [8, 82], so the prevalence of these, and other post-translational genes, suggests these genes may also represent adaptations by soil oligotrophs to resource limitation.

## Conclusions

Our analyses of three independent datasets suggest that there are particular bacterial taxa that are consistently associated with more carbon-limited environments. However, we also identified a wide range of taxonomic groups that include members with distinct, or inconsistent, life history strategies. Likewise, there were relatively few genomic attributes that were consistently associated with taxa inferred to be either copiotrophic or oligotrophic. Several of the hypotheses regarding the attributes of oligotrophic bacteria, hypotheses derived from previous studies, were supported by our analyses, including our finding that soil oligotrophic bacteria typically had smaller genomes, lower maximum potential growth rates, enrichment for gene pathways that confer metabolic flexibility, and more genes associated with post-translational processes. However, we note that many of the functional genes and genomic attributes that we found to differ between inferred oligotrophs and copiotrophs were not consistent across the three datasets, suggesting that there is a diverse array of ecological strategies used by soil bacteria to cope with reduced carbon availability. There is no single way to be an oligotroph.

## Supplementary Material

Oligotrophy_SI-ISMECommunications_ycae081

Dataset_S1-Oligotrophy-ISMECommunications_ycae081

Dataset_S2-Oligotrophy-ISMECommunications_ycae081

Dataset_S3-Oligotrophy-ISMECommunications_ycae081

Dataset_S4-Oligotrophy-ISMECommunications_ycae081

Dataset_S5-Oligotrophy-ISMECommunications_ycae081

## Data Availability

All data used in this study are available in the main text or the supplementary material. Raw 16S rRNA sequences for the “rhizosphere” dataset can be found on the European Nucleotide Archive under accession number PRJEB25694 for bulk soils and PRJEB25692 for rhizosphere soils. Raw 16S rRNA gene sequencing data from the “soil profile” dataset can be found on Figshare at https://doi.org/10.6084/m9.figshare.4702711. Raw 16S rRNA sequence from “microcosm” samples can be found on the European Nucleotide Archive under accession number PRJNA1071192.
