# Supplementary material for: Taxonomic and genomic attributes of oligotrophic soil bacteria
Source: ISME Commun. 2024 Jun 12;4(1):ycae081. doi: 10.1093/ismeco/ycae081 (PMC11234899; doi:10.1093/ismeco/ycae081)
Supplement: Oligotrophy_SI-ISMECommunications_ycae081 [file oligotrophy_si-ismecommunications_ycae081.docx]

*ISME Communications*

Supporting information for

**The taxonomic and genomic attributes of oligotrophic soil bacteria**

Nicholas B. Dragone^1*^, Michael Hoffert^1,2^, Michael S. Strickland^3^, Noah Fierer^1,2*^

^1^Cooperative Institute for Research in Environmental Science, University of Colorado Boulder, Boulder, CO, US

^2^Department of Ecology and Evolutionary Biology, University of Colorado Boulder, Boulder, CO, US

^3^Department of Soil and Water Systems, University of Idaho, Moscow, ID, US

**Contents of this file**

Table S1

Fig. S1 to S4

Captions for Dataset S1 – S5

SI References 1 - 11

**Additional Supporting Information (files uploaded separately)**

Datasets S1 – S5

**Summary**

The supporting information in this file contains tables, figures, and captions for attached

datasets referenced in the main paper. All processing steps used to generate these data

are described in the methods. More specifically this SI provide details about the taxa found to be associated with carbon rich and carbon limited environments, the genomes identified as more oligotrophic and copiotrophic, and the annotated genes found in greater abundance in more oligotrophic and copiotrophic genomes.

| **Gene or Gene Category** | **Associated COGs** | **Reference** |
| --- | --- | --- |
| Amino acid transport and metabolism | COG category E | [1] |
| Chemotaxis and motility | COG category N | [2, 3] |
| Lipid transport and metabolism | COG category I | [3] |
| Secondary metabolite biosynthesis, transport, metabolism | COG category Q | [ 3] |
| Defense mechanisms | COG category V | [4] |
| Transcription | COG category K | [4] |
| Signal transduction | COG category T | [4] |
| Cellular replication, recombination, repair | COG category L | [5] |
| Glycine betaine ABC transporter (ProX) | COG2113, COG3760, COG2113, COG3760 | [6] |
| RNA polymerase, extracytoplasmic E (rpoE) | COG1595, COG3343, COG5503 | [3] |
| Trehalose synthase and transporter | COG3281, COG4813 | [7] |
| Form 1 CO dehydrogenases (coxL) | COG1529 | [8] |
| [NiFe] hydrogenases | COG0374, COG0680, COG1740, COG1969, COG3260, COG3261, COG3262 | [9] |
| Thiamine biosynthesis | COG2145, COG0301, COG0351, COG0352, COG0422, COG0476, COG0611, COG1060, COG1564, COG2022, COG2104 | [2] |
| Poly-B-hydroxybutyrate, polyhydroxyalkanoate | COG3243, COG3937, COG5394, COG5490 | [10] |

**Table S1**: The COGs and COG categories associated with each hypothesis outlined in Table 1.

*
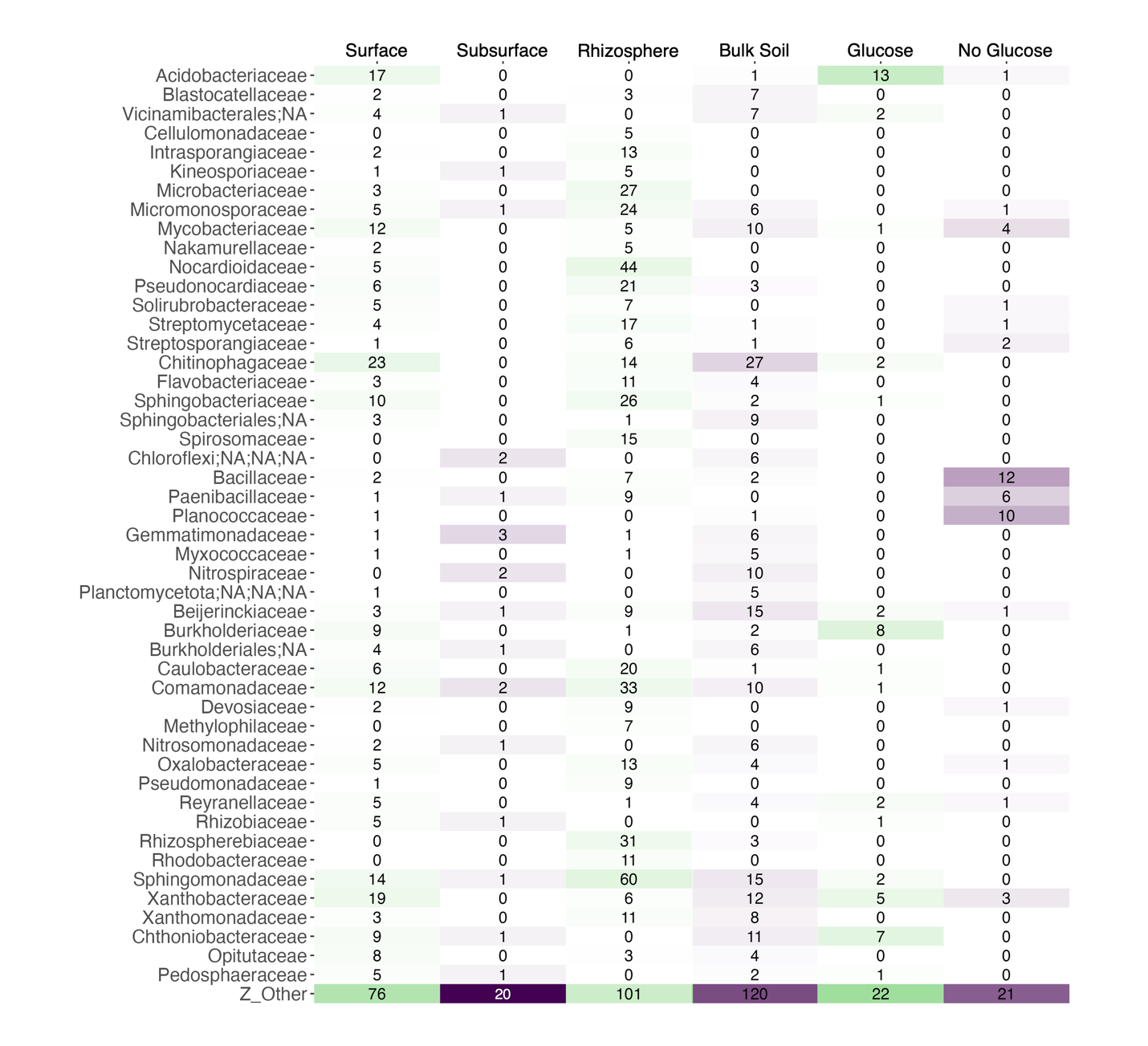
*

**Fig. S1**: Heatmap displaying the number of GTDB genomes identified as associated with surface (303), subsurface (40), bulk soils (336), rhizosphere (592), glucose (71), and no glucose (66) soils. Genomes associated with the rhizosphere and the surface soils are considered more copiotrophic while those associated with bulk soils and the subsurface soils are considered more oligotrophic. Genomes are grouped by phyla and are ordered alphabetically by family. If no family-level information is available, the most specific taxonomic information available is displayed. Information about these genomes can be found in Dataset S2.

**
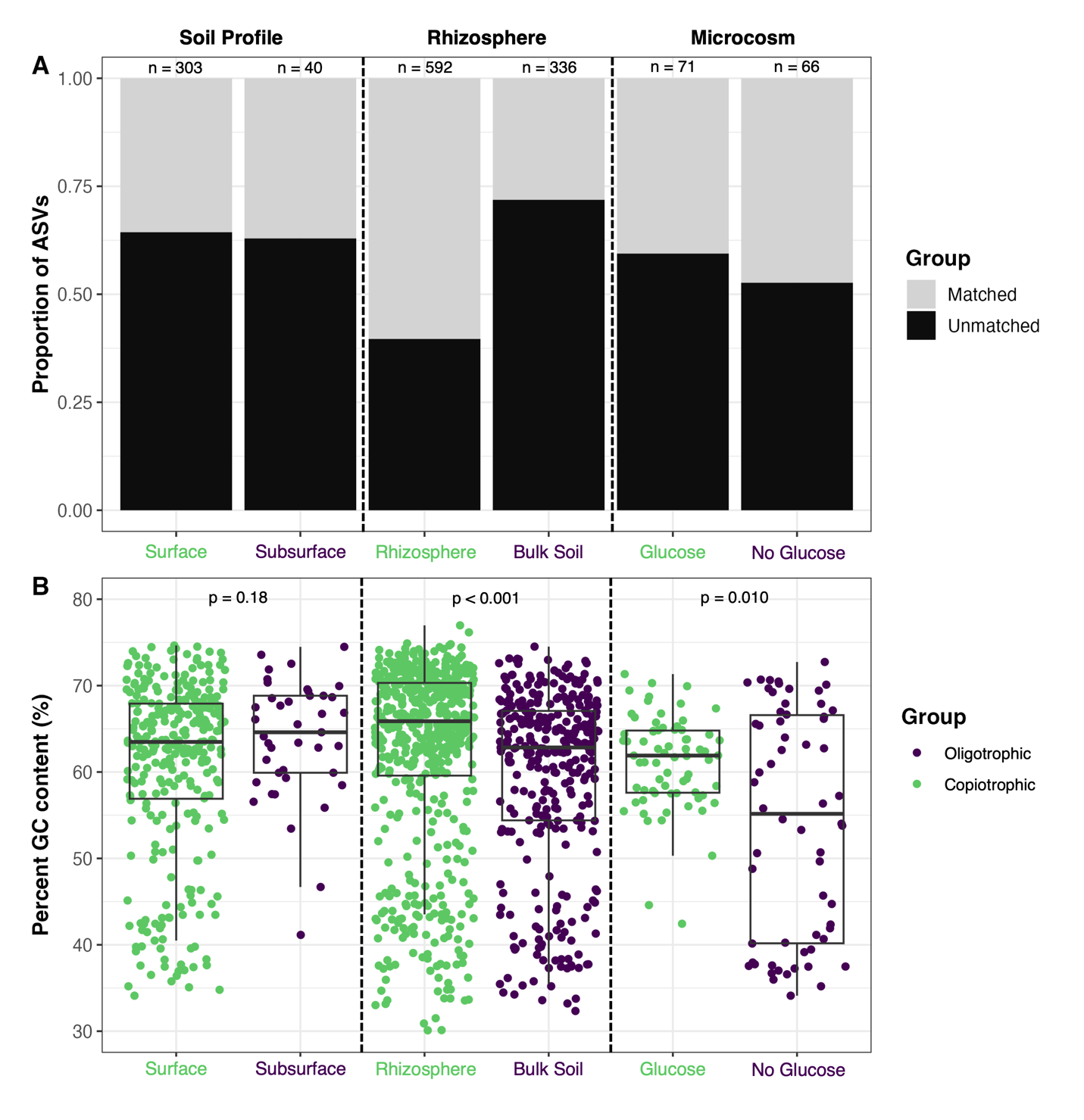
**

**Fig S2:** Additional genomic characteristics of the surface, subsurface, rhizosphere, bulk soil, glucose, and no glucose genomes. **A)** The proportion of the surface ASVs (n=1271), subsurface ASVs (n=178), rhizosphere ASVs (n=1366), bulk soil ASVs (n=2779), glucose ASVs (n=97), and no glucose (n=80) that matched to GTDB genomes (see methods and Dataset S3 for more details). **B)** There was no significant difference in the percent GC content between the surface and subsurface genomes (Mann-Whitney U, p = 0.18) but the GC percentage was significantly higher in the rhizosphere compared to the bulk soil genomes (Mann-Whitney U, p < 0.001) and significantly higher in the glucose genomes compared to the no glucose genomes (Mann-Whitney U, p = 0.010). Raw data is presented in Dataset S2.


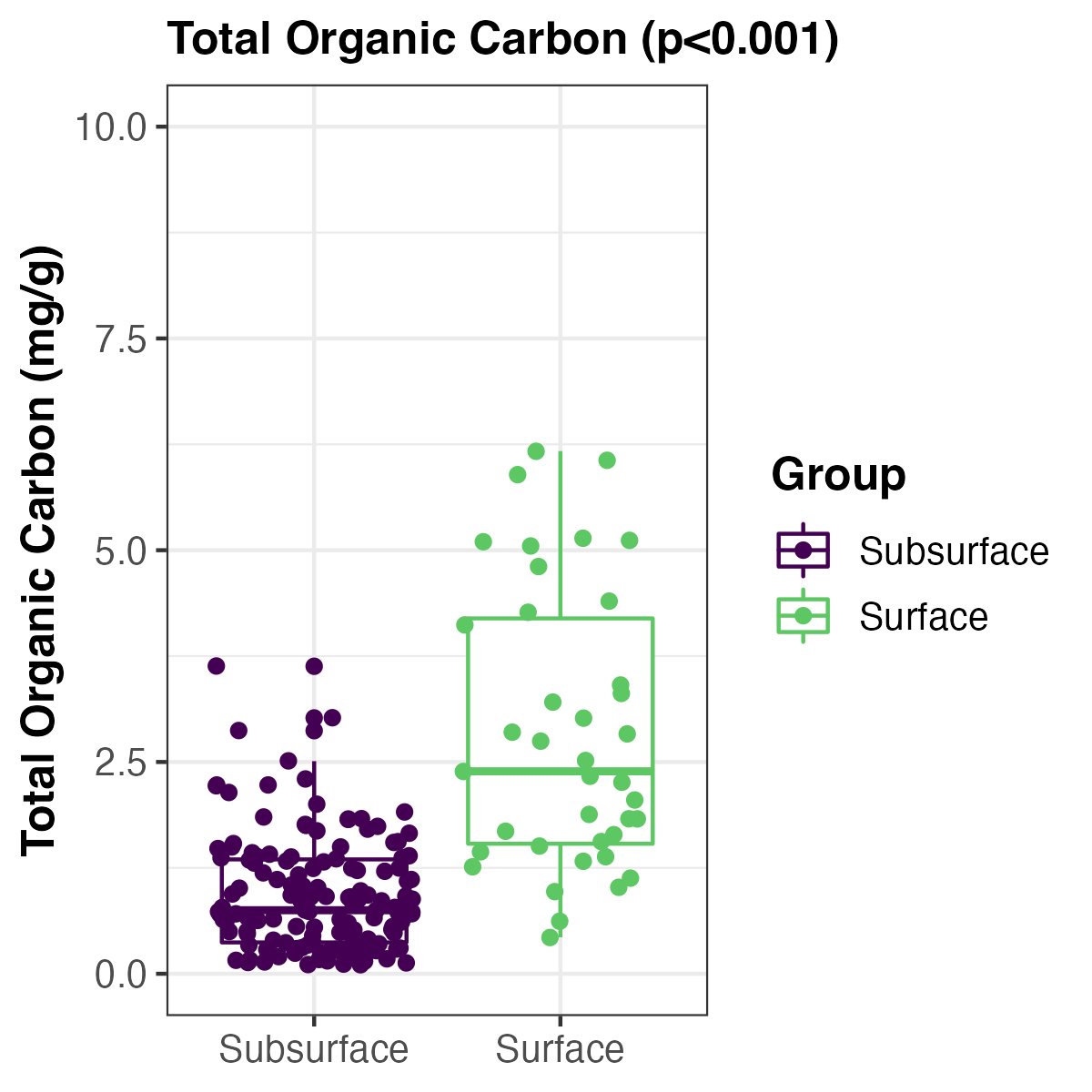


**Fig. S3**: Total organic carbon in the surface (n=39) and subsurface (n=139) soil samples. Surface soils were collected from 0 – 20cm depth while subsurface soils were collected >*2*0cm in depth. Subsurface samples had significantly lower organic carbon concentrations than the surface soils (Mann-Whitney U, p <0.001). For more information about organic carbon, see Brewer et al. [11].

*
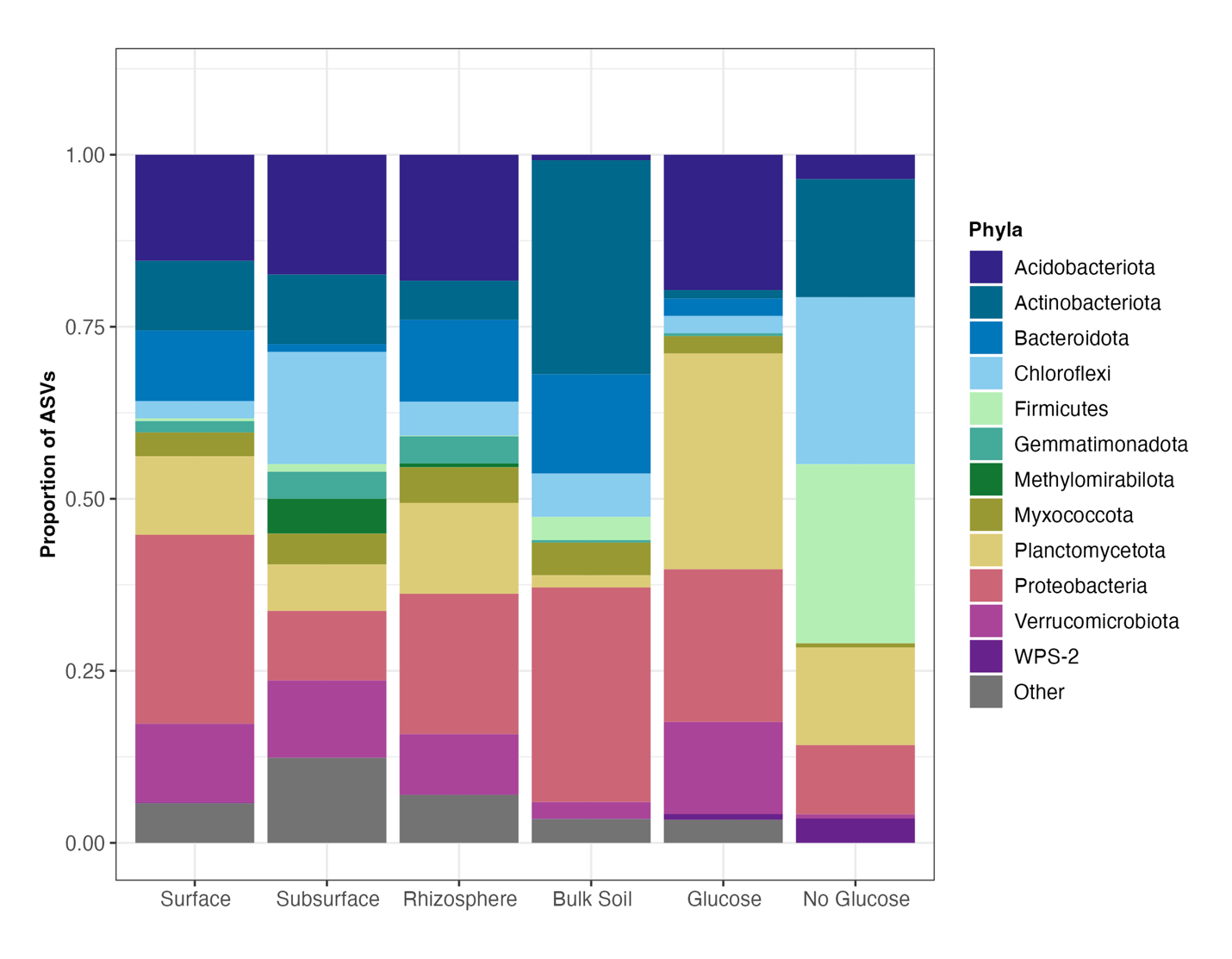
*

**Fig. S4**: Overview of the bacterial taxa associated with surface (1271 ASVs), subsurface (178 ASVs), rhizosphere (1366), bulk soil (2779 ASVs), glucose (239 ASVs), and no glucose (169 ASVs) soils. The size of the bar represents the proportion of the total number of ASVs assigned to the phylum. More specific taxonomic information can be found in Fig. 1 and Dataset S2.

**Dataset S1: (Separate File, Dataset_S1_Oligotrophy.xlsx)**

Table of all ASVs recovered from amplicon sequencing of the 16S rRNA gene for **A)** the ‘soil profile’ dataset, **B)** the ‘rhizosphere’ dataset, and **C)** the ‘microcosm’ dataset. Taxonomic classification of each ASV and the 16S rRNA sequence for that ASV are also included.

**Dataset S2: (Separate File, Dataset_S2_Oligotrophy.xlsx)**

**A)** Table of all ASVs identified as being associated with surface soils or subsurface soils in the ‘soil profile’ dataset. **B)** Table of all ASVs identified as being associated with either the rhizosphere soils or bulk soils in the ‘rhizosphere’ dataset. **C)** Table of all ASVs identified as being associated with either the glucose soils or no glucose soils in the ‘microcosm’ dataset. Predicted taxonomy of each ASV and the statistical significance (p value) is also presented. See methods for more information on how ASVs are assigned to each category.

**Dataset S3: (Separate File, Dataset_S3_Oligotrophy.xlsx)**

GTDB genomes that were found to be associated with **A)** surface soils and subsurface soils, **B)** rhizosphere soils and bulk soils, and **C)** glucose amended soils and unamended ‘no glucose’ soils. More information about how genomes were found to be associated with a specific environment can be found in the methods. For all genomes, information about the genome type (MAG vs. Isolate), completeness, contamination, gc percentage, estimated genome size, predicted taxonomy, and estimated minimum doubling time are presented. If a value was not measured or could not be found it is represented by “NA.”

**Dataset S4: (Separate File, Dataset_S4_Oligotrophy.xlsx)**

The abundance of all 4877 COGs in each genome associated with the **A)** ‘soil profile’ dataset, **B)** the ‘rhizosphere’ dataset, and **C)** the ‘microcosm’ dataset. The abundance of each COG in a specific genome has been normalized based on the estimated length of that genome (see Dataset S3 and methods for more information).

**Dataset S5: (Separate File, Dataset_S5_Oligotrophy.xlsx)**

The COGs found to be consistently more abundant in copiotrophic genomes and oligotrophic genomes. A COG was found to be associated with copiotrophs or oligotrophs if the abundance (normalized reads per million) was found to be significantly more abundant in that group in across the ‘rhizosphere’, ‘bulk soil’, and ‘microcosm’ datasets. See methods for more information about these classifications. Provided COG classifications are based on the database of Clusters of Orthologous Genes (COGs) ontology v.2020.

**References:**

1. Qin Q-L, Li Y, Sun L-L, Wang Z-B, Wang S, Chen X-L, et al. Trophic specialization results in genomic reduction in free-living marine Idiomarina bacteria. mBio. 2019; 10:e02545-18.

2. Roller BRK, Stoddard SF, Schmidt TM. Exploiting rRNA operon copy number to investigate bacterial reproductive strategies. Nat Microbiol. 2016; 1:1–7.

3. Lauro FM, McDougald D, Thomas T, Williams TJ, Egan S, Rice S, et al. The genomic basis of trophic strategy in marine bacteria. Proc Natl Acad Sci USA. 2009; 106:15527–15533.

4. Dutta C, Paul S. Microbial lifestyle and genome signatures. Nat Prod Rep. 2012; 13:153–162.

5. Koch AL. Oligotrophs versus copiotrophs. BioEssays 2001; 23:657–661.

6. Noell SE, Giovannoni SJ. SAR11 bacteria have a high affinity and multifunctional glycine betaine transporter. Environ Microbiol. 2019; 21:2559–2575.

7. Bird JT, Tague ED, Zinke L, Schmidt JM, Steen AD, Reese B, et al. Uncultured microbial phyla suggest mechanisms for multi-thousand-year subsistence in Baltic Sea sediments. mBio. 2019; 10:e02376-18.

8. Cordero PRF, Bayly K, Man Leung P, Huang C, Islam ZF, Schittenhelm RB, et al. Atmospheric carbon monoxide oxidation is a widespread mechanism supporting microbial survival. ISME J. 2019; 13:2868–2881.

9. Greening C, Carere CR, Rushton-Green R, Harold LK, Hards K, Taylor MC, et al. Persistence of the dominant soil phylum Acidobacteria by trace gas scavenging. Proc Natl Acad Sci USA. 2015; 112:10497–10502.

10. Poindexter JS. Oligotrophy. In: Alexander M (ed). Advances in Microbial Ecology. Springer US, Boston, MA, 1981. pp 63–89.

11. Brewer TE, Aronson EL, Arogyaswamy K, Billings SA, Botthoff JK, Campbell AN, et al. Ecological and genomic attributes of novel bacterial taxa that thrive in subsurface soil horizons. mBio. 2019; 10:e01318-19.
